# Supplementary material for: Replication stress induces POLQ-mediated structural variant formation throughout common fragile sites after entry into mitosis
Source: Nat Commun. 2024 Nov 6;15:9582. doi: 10.1038/s41467-024-53917-8 (PMC11541566; doi:10.1038/s41467-024-53917-8)
Supplement: Supplementary file 1 — Supplementary Information [file 41467_2024_53917_MOESM1_ESM.pdf]

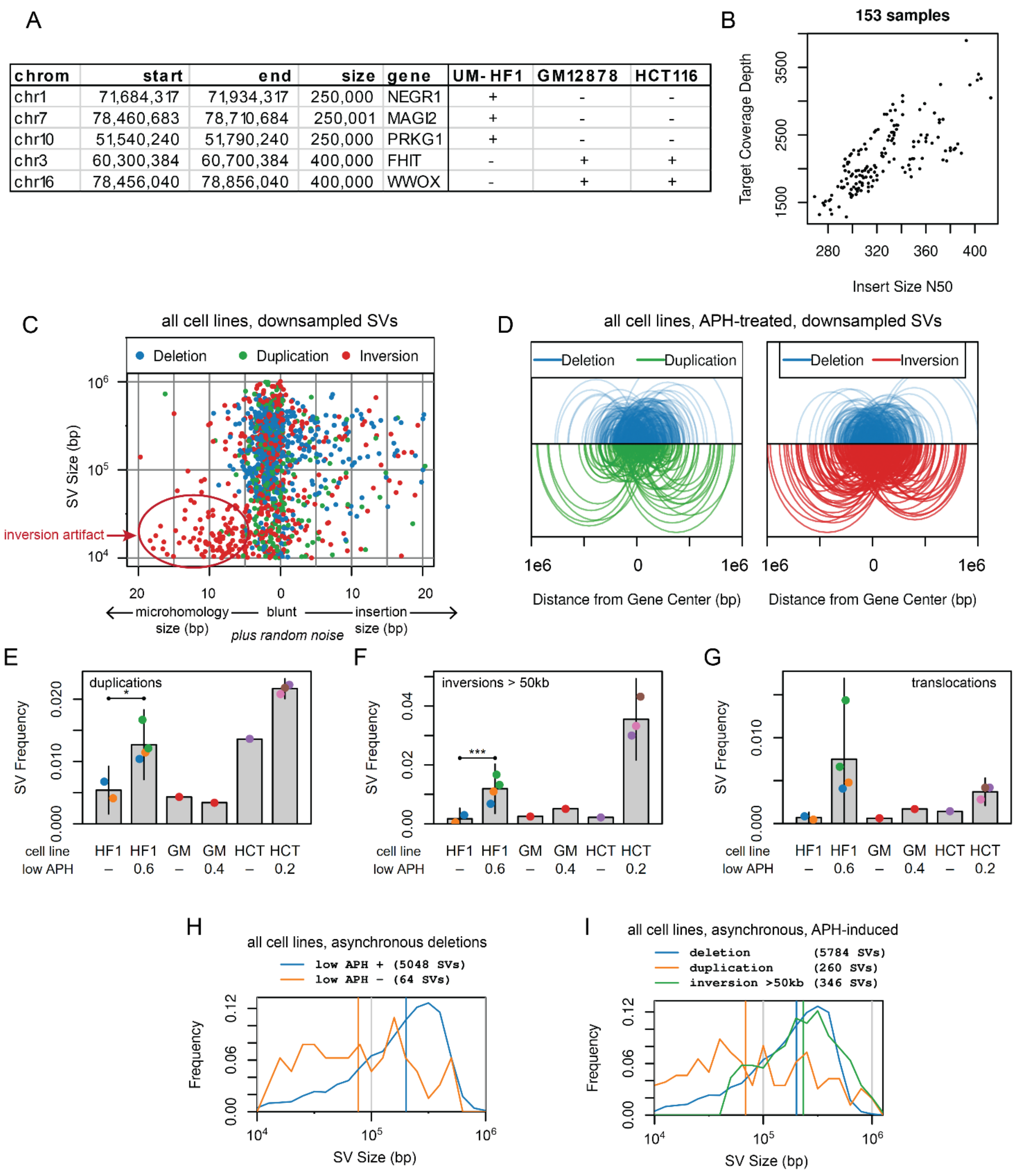


| chrom | start | end | size | gene | UM-HF1 | GM12878 | HCT116 |
|---|---|---|---|---|---|---|---|
| chr1 | 71,684,317 | 71,934,317 | 250,000 | NEGR1 | + | - | - |
| chr7 | 78,460,683 | 78,710,684 | 250,001 | MAGI2 | + | - | - |
| chr10 | 51,540,240 | 51,790,240 | 250,000 | PRKG1 | + | - | - |
| chr3 | 60,300,384 | 60,700,384 | 400,000 | FHIT | - | + | + |
| chr16 | 78,456,040 | 78,856,040 | 400,000 | WWOX | - | + | + |

**Supplementary Figure 1. svCapture profiles in asynchronous human cell lines.**

**A.** svCapture target regions used in different cell lines ("+" symbols).

**B.** Summary of sequencing library insert sizes, expressed as N50, and target region coverage for all independent biological replicates in the study (n = 153).

**C.** Correlation of asynchronous sample SV size to junction insert/microhomology by SV type. Each type is downsampled to no more than the inversion event count (n = 456) to compare distributions. Circled small inversions (red) with large microhomologies are a known library artifact resulting from intramolecular hybridization and synthesis during end-filling of Tn5-cleaved ends. Accordingly, only inversions >50kb are included in subsequent figures.

**D.** Arcs connect the two breakpoint positions of up to 500 downsampled individual asynchronous SVs of each type. Color intensity reflects the fraction of SVs at that size to emphasize that deletions (blue) arise more central to CFS genes whereas duplications (green) and inversions (red) have a higher fraction of flanking events.

**E to G.** Duplication, inversion, and inter-target translocation SV induction, respectively, for the same data sources as Figure 1E. Note the Y-axis limits and lower fold induction as compared to deletions. Error bars are mean +/- 2SD. HF1 p-values from two-sided negative binomial generalized linear model: duplication, 0.00534; inversion, 3.29e-05. *, p <= 0.01; **, p <= 0.001; ***, p <= 0.0001.

**H.** SV size distributions merging asynchronous cultures of all cell lines, for the same data sources as Figure 1F, stratified by low-dose APH treatment to show the larger size of induced (blue) vs. spontaneous (orange) deletions.

**I.** SV size distributions from asynchronous, APH-treated cells, for the same data sources as H, stratified by SV type to show the smaller size of duplications (orange) relative to deletions (blue) and inversions (green). With lower fold induction, more duplications are expected to have arisen prior to APH treatment.

Source data are provided as a Source Data file.

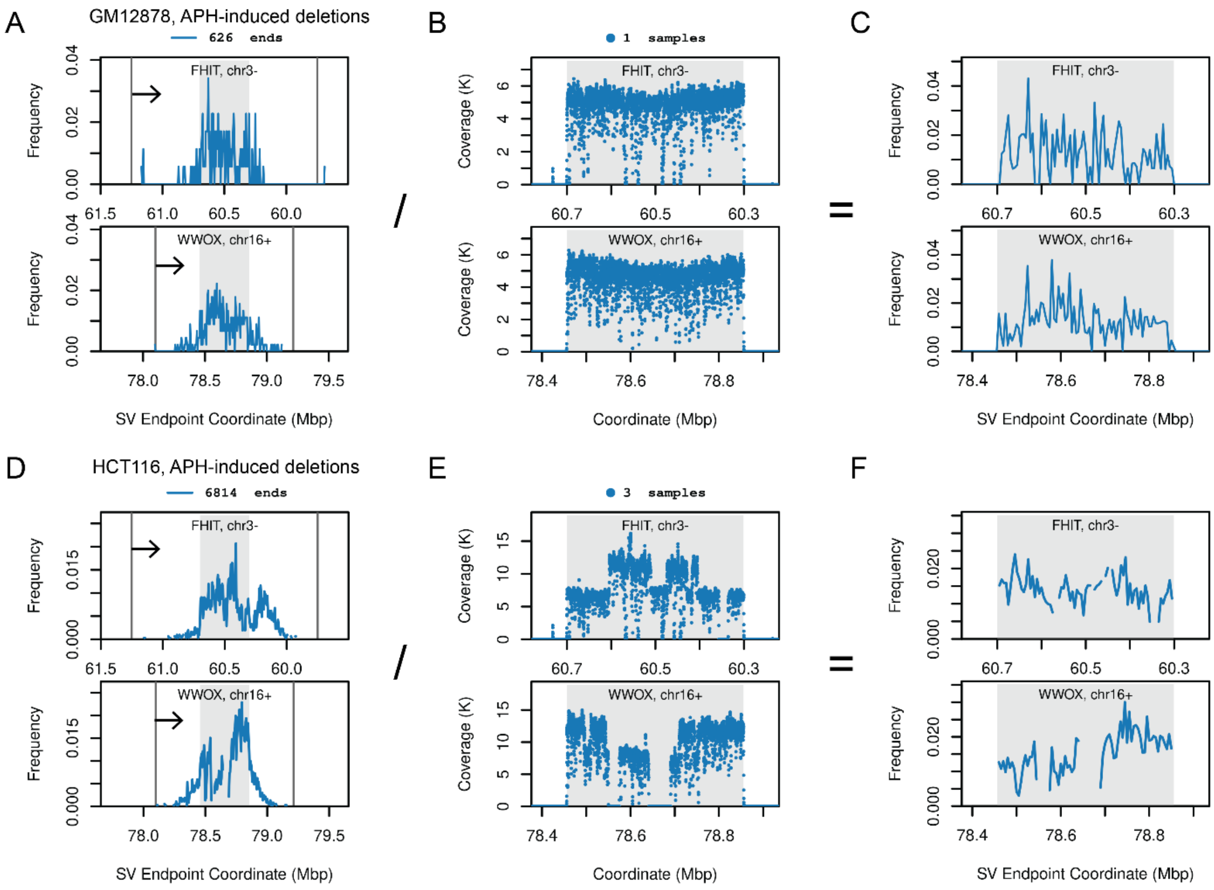


**Supplementary Figure 2. SV breakpoint locations in asynchronous human cell lines.**

**A to C.** Placement of GM12878 APH-induced deletion breakpoints (A and C) and read coverage (B) in the *FHIT* and *WWOX* target genes, analogous Figure 1G to 1I for HF1 cells.

**D to F.** Like panels A to C, now for HCT116 cells. Panels C and F show breakpoint distributions from panels A and D normalized to read coverage >= 500 from panels B and E. Panel E reveals clonal baseline SVs in HCT116 at *FHIT* and *WWOX*, consistent with prior genomic analysis of this cell line. Additional de novo SVs still occur superimposed onto the alleles carrying these baseline SVs.

Source data are provided as a Source Data file.

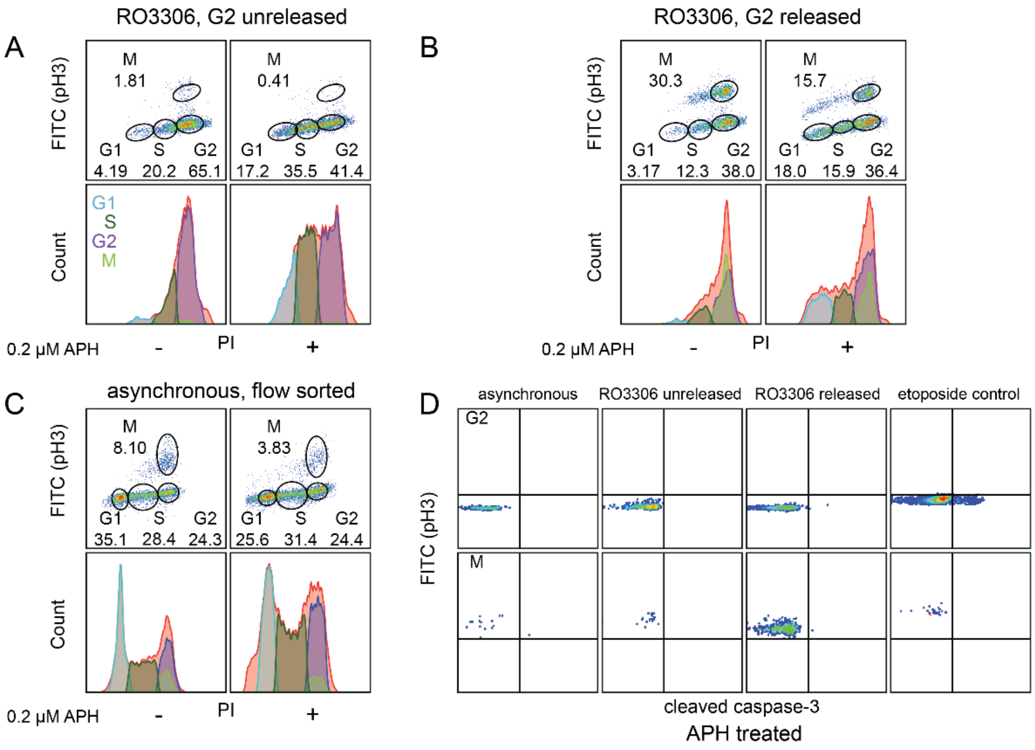


**Supplementary Figure 3. Isolation and viability of S, G2, and M-phase cells.**

**A.** Example HCT116 flow sort for the synchronization paradigm with G2 cells collected prior to release from RO3306. x-axis, DNA content; y-axis, pH3. Lower panels show cell cycle distributions, revealing synchronization and the impact of APH on S-phase accumulation.

**B.** Like A, now for the RO3306 synchronization paradigm with G2 cells collected after release.

**C.** Like A, now for HCT116 cells flow sorted by cell cycle phase from cultures without RO3306.

**D.** Flow cytometry plots of cleaved caspase 3, x-axis, and pH3, y-axis, showing that G2 cells remained viable during APH treatment, arrest, and release. The rightmost panel was treated with 10µM etoposide as a positive control.

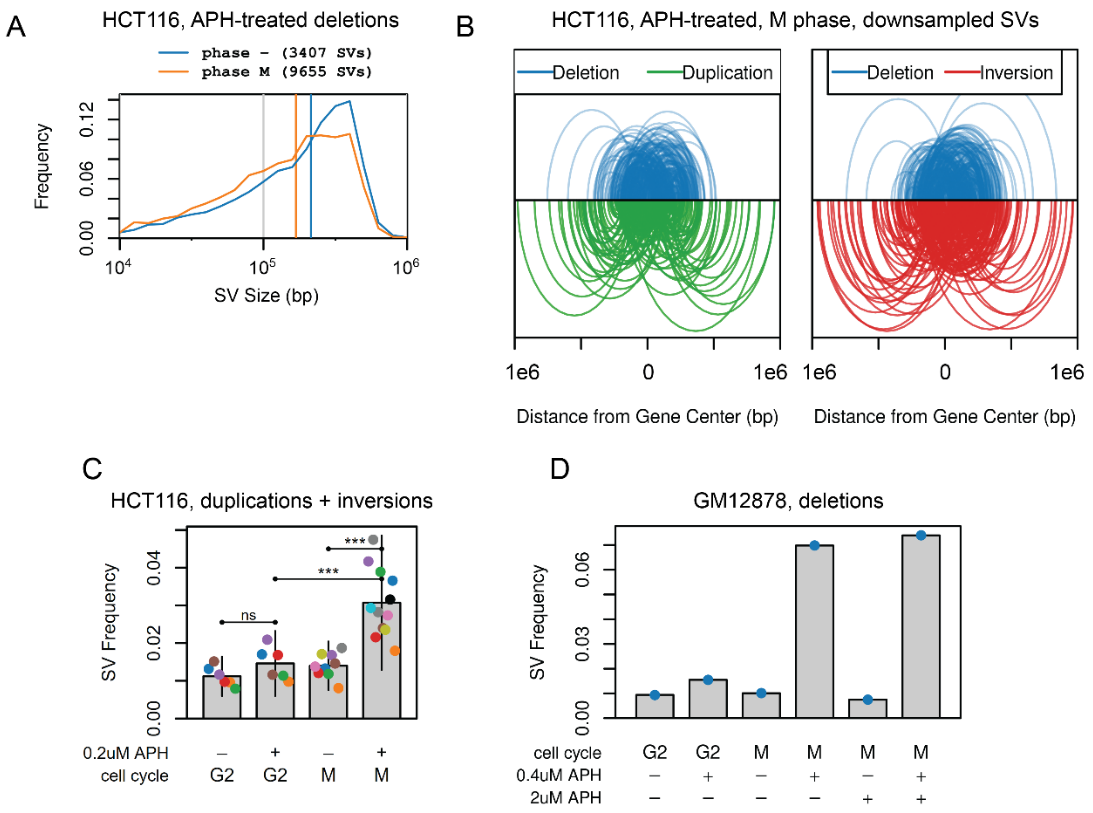


**Supplementary Figure 4. APH induces SV junction formation during mitosis.**

**A.** Similar SV length distributions for APH-induced deletions in asynchronous (-) vs. M-phase HCT116 cells. Independent biological replicate (SV) numbers by cell cycle type: asynchronous, 3 (3407); M, 13 (9655).

**B.** Downsampled SVs like Figure S1D, showing that deletions (blue) induced in M-phase HCT116 cells tend to be more gene-central as compared to duplications (green) and inversions (red).

**C.** Duplication plus inversion frequencies as a function of cell cycle phase in HCT116 cells under the synchronization and release paradigm of Figure 2A. Plot elements and statistics are the same as Figure 2D. Independent biological replicate (SV) numbers by cell cycle/APH are: G2/-, 6 (128); G2/+, 6 (150); M/-, 9 (261); M/+, 12 (763). P-values are: G2/- vs. G2/+, 0.035; G2/+ vs. M/+, 9.36e-13; M/- vs. M/+, 1.49e-19.

**D.** SV frequencies from GM12878 cells harvested with and without high-dose APH suppression of MiDAS under the synchronization and release paradigm of Figures 2G and 2K. This single replicate experiment matches results from HCT116.

Source data are provided as a Source Data file.

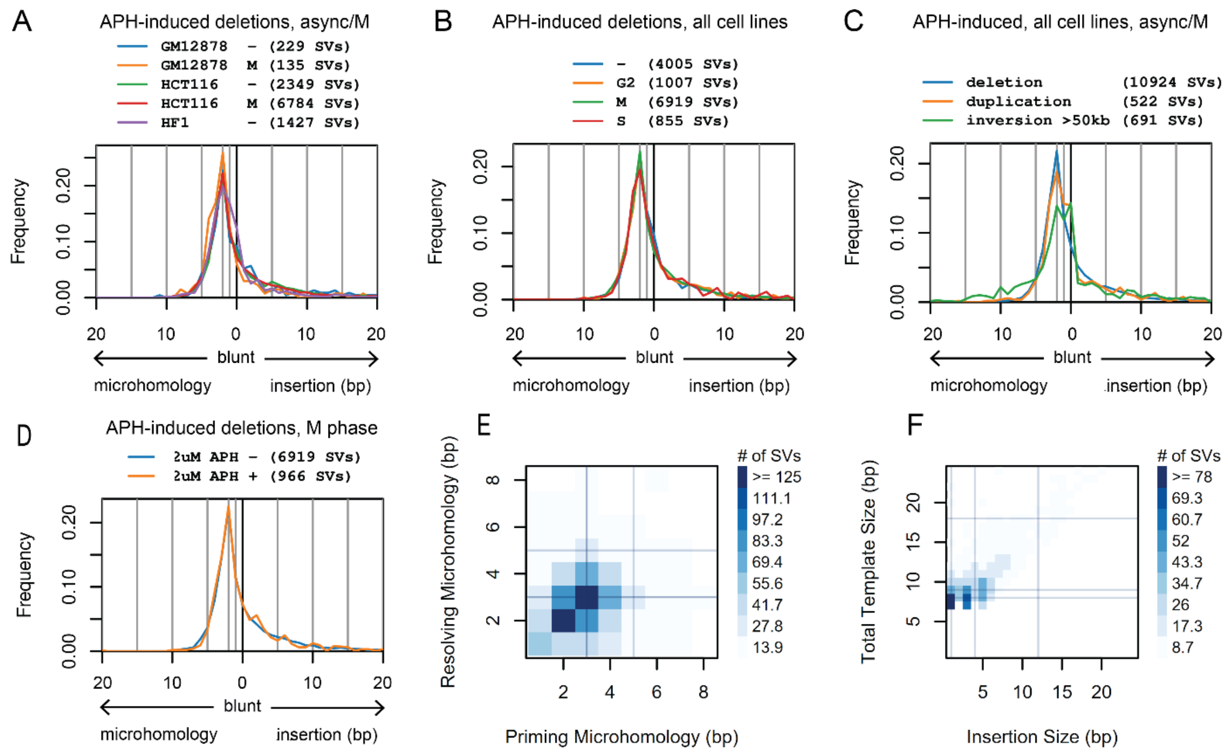


**Supplementary Figure 5. Junction analysis for microhomologies and templated insertions.**

**A.** Distribution of microhomology and insert sizes by cell line and cell cycle phase (async/- vs. M), for the same data sources as Figure 4C. SV counts are shown above plots.

**B.** Like panel A, now stratified by cell cycle phase over APH-treated samples from all cell lines.

**C.** Like panel A, now stratified by SV type detected in asynchronous or M-phase cells for all APH-treated samples.

**D.** Like panel A, now showing APH-induced deletions in M-phase by concurrent high-dose APH treatment. Distributions throughout are nearly identical, except for inversions that likely retain a small amount of known artifact mechanisms unique to that SV type.

**E.** Correlated size distributions of priming and resolving microhomologies over all insertion templates.

**F.** Correlated insert and total template (including flanking microhomologies) size distributions. A total of 1099 insertion SVs with identified templates are plotted in E and F from the same data sources as Figure 1F.

Source data are provided as a Source Data file.

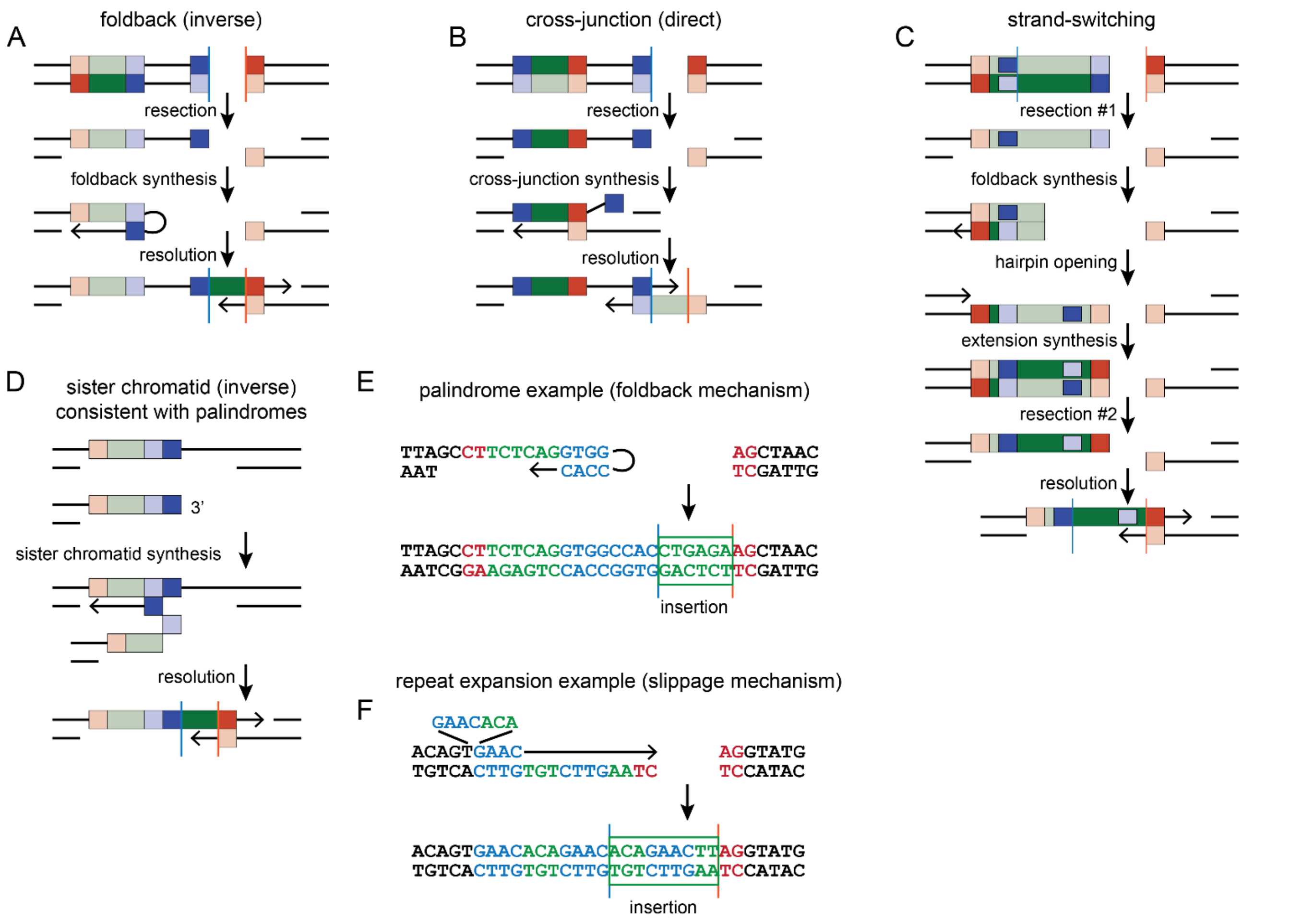


**Supplementary Figure 6. Mechanisms of templated insertion.**

**A** to **C.** Previously described templated insertion mechanisms. Only left-sided templates are drawn; right-sided templates are flipped but retain the same strandedness, e.g., right-sided foldback templates are also on the bottom strand. The strand-switching mechanism was not readily observed in our data.

**D.** An alternative method for generating inverse insertions using the sister chromatid as template, which is compatible with palindromic priming sequences.

**E.** Observed example of a small subset of inverse events where the breakpoint mapped to a perfect palindrome, which alters the placement of the called junction position as compared to non-palindromic foldbacks. If such insertions occur by foldback synthesis as drawn, the entire palindrome could not anneal in the initiating hairpin.

**F.** Observed example of an expansion-class insertion, drawn here for the slippage formation model. Base colors and boxes in E and F follow Figure 4A.

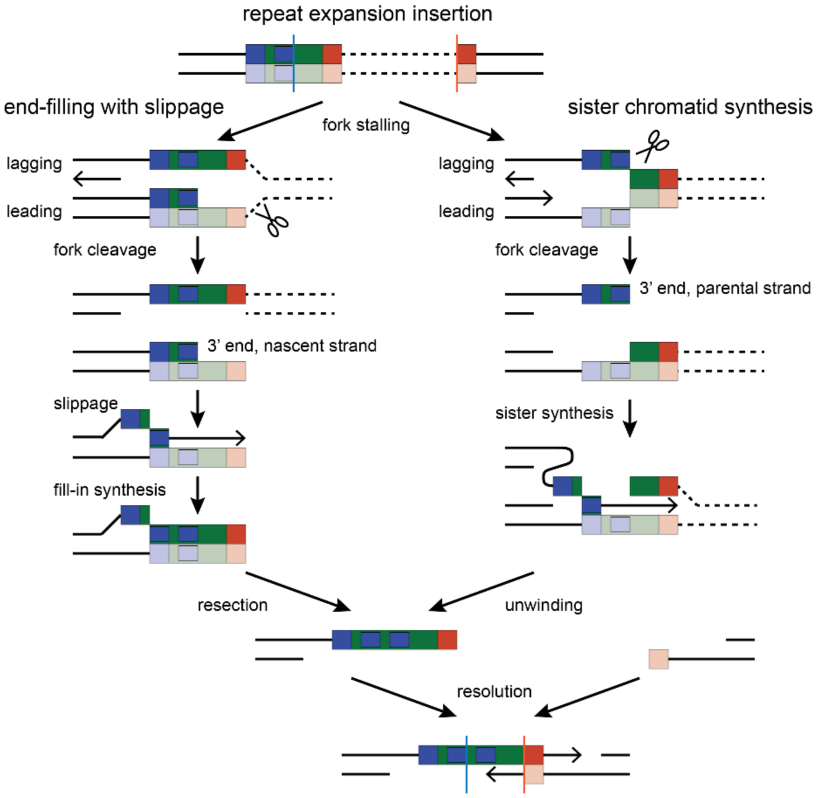


**Supplementary Figure 7. Possible mechanisms of SV junction repeat expansion.**

Two models illustrating how repeat expansion insertion might occur during the process of SV junction formation. The two models differ in the orientation and location of replication fork cleavage and therefore whether a parental or nascent DNA strand acts as the primer terminus. The models are similar in that the insertion template is found on the leading strand template.

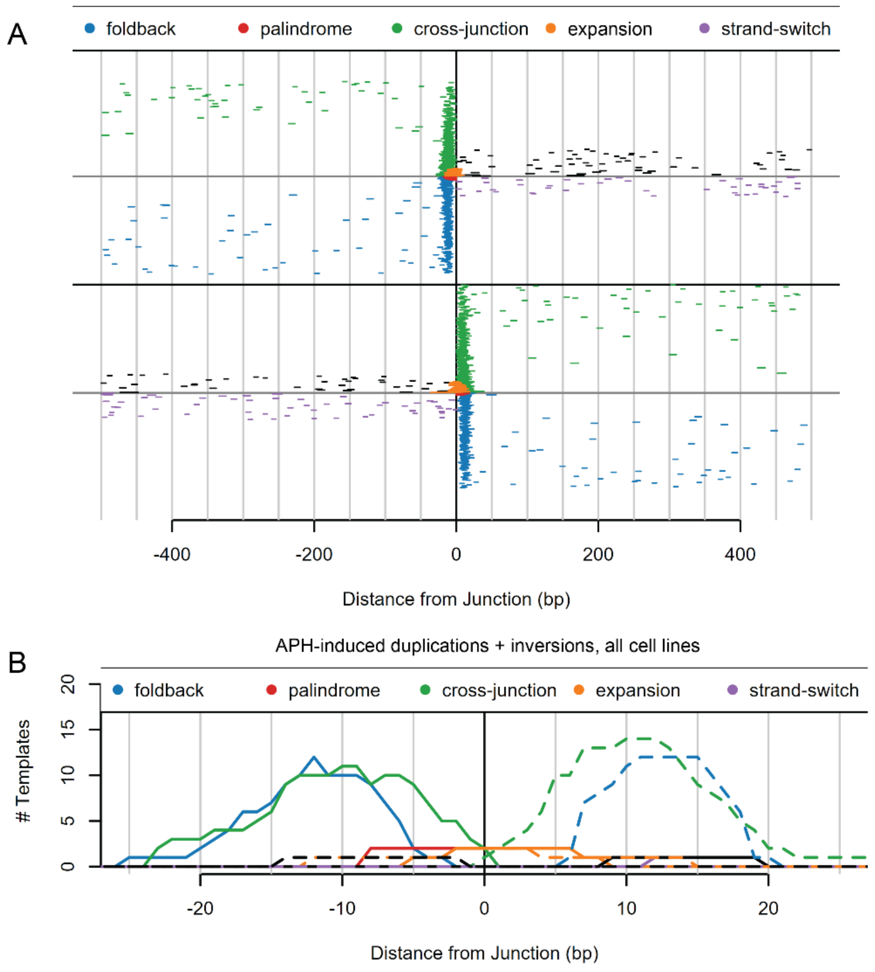


**Supplementary Figure 8. Locations of insertion templates.**

**A.** Wide view of insertion template locations for APH-induced deletions throughout the 1kb interrogation regions, the same data as plotted in Figure 4F. Randomly distributed candidate templates further from breakpoints likely represent fortuitous matches unrelated to junction formation mechanisms.

**B.** Bases contributing to insertion templates at APH-induced duplication and inversion SVs across all cell lines, showing a similar template location pattern as for deletions. Data sources are the same as Figure 4G.

Source data are provided as a Source Data file.

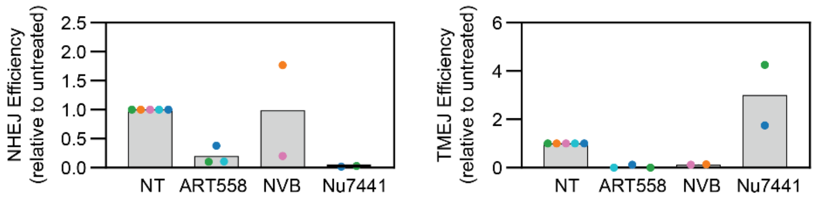


**Supplementary Figure 9. DSB repair inhibition.**

Transfected oligonucleotide assay assessing inhibition of NHEJ (left) and TMEJ (right) by chemical inhibitors in wild-type (non-DSB repair KO) HCT116 cells. Sample point colors denote experimental batches. Number of independent biological replicates by inhibitor: NT, 5; ART558, 3; NVB, 2; Nu7441, 2. Grey bars: mean.

Source data are provided as a Source Data file.

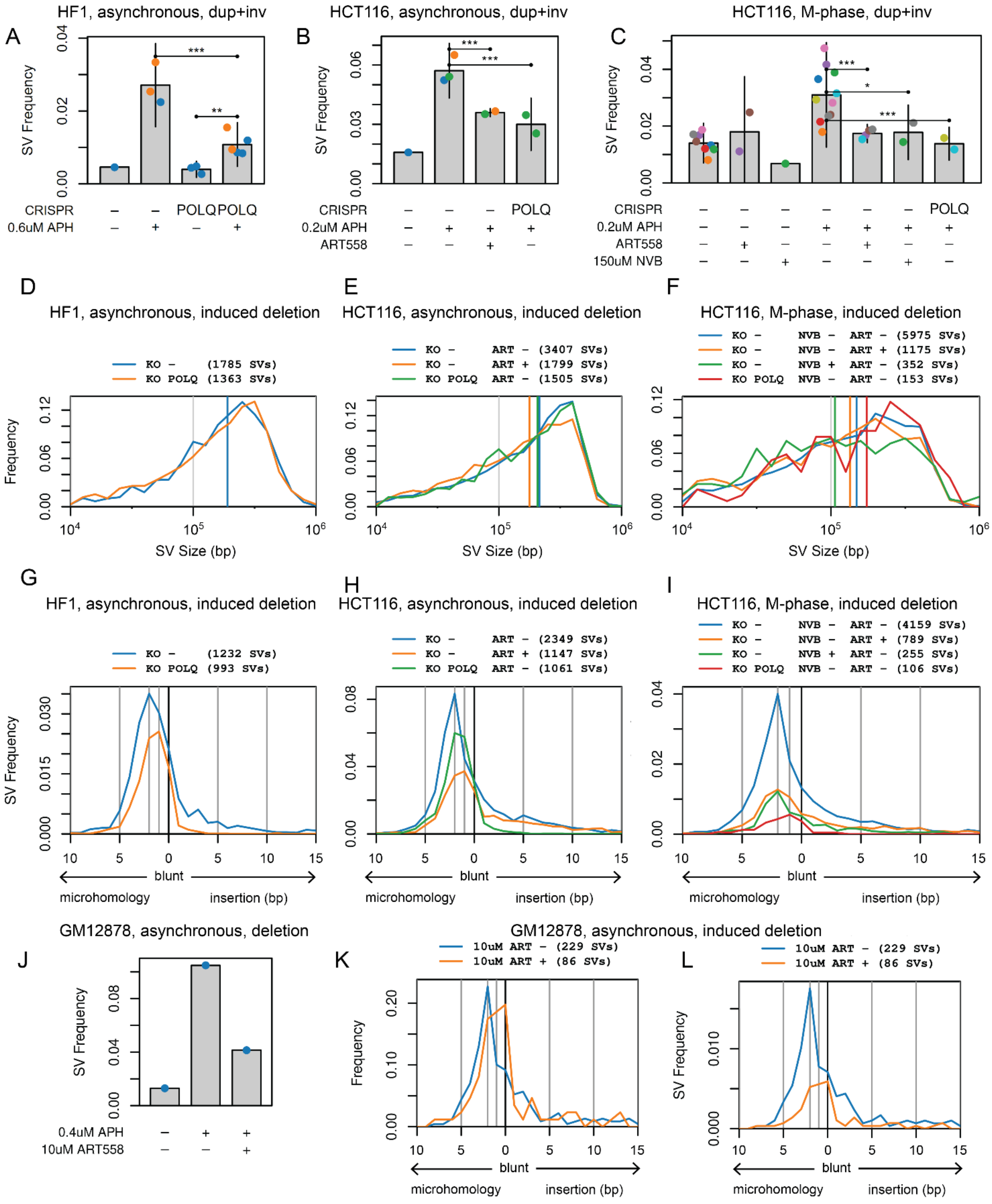


**Supplementary Figure 10. POLQ role in SV formation at CFSs.**

**A.** HF1 asynchronous duplication plus inversion SV frequency with POLQ KO. Sample point colors denote experimental batches. Error bars are mean +/- 2 SD of two or more independent biological

replicates. Replicate (total SV) numbers by CRISPR/APH are: -/-, 1 (10); -/+, 3 (193); POLQ/-, 3 (26); POLQ/+, 5 (117). P-values from two-sided negative binomial generalized linear model: -/+ vs. POLQ/+, 8.05e-12; POLQ/- vs. POLQ/+, 1.58e-04. *, p <= 0.01; **, p <= 0.001; ***, p <= 0.0001.

**B.** HCT116 asynchronous duplication plus inversion SV frequency with POLQ KO and inhibition by ART558. Plot labeling and statistics are the same as A. Replicate (total SV) numbers by CRISPR/APH/ART558 are: -/-/-, 1 (22); -/+/-, 3 (350); -/+/+, 2 (176); POLQ/+/-, 2 (147). P-values are: -/+/- vs. -/+/+, 1.83e-07;-/+/- vs. POLQ/+/-, 7.38e-07.

**C.** HCT116 M-phase duplication plus inversion SV frequency with POLQ KO and inhibition by ART558 and novobiocin (NVB). Plot labeling and statistics are the same as A. Replicate (total SV) numbers by CRISPR/APH/ART558/NVB are: -/-/-/-, 8 (234); -/-/+/-, 2 (68); -/-/-/+, 1 (9); -/+/-/-, 11 (709); -/+/+/-, 4 (167); -/+/-/+, 2 (92); POLQ/+/-/-, 2 (67). P-values are: -/+/-/- vs. -/+/+/-, 2.26e-06; -/+/-/- vs. -/+/-/+, 6.83e-03; -/+/-/- vs. POLQ/+/-/-, 4.81e-08.

**D** to **F.** APH-induced deletion SV size distributions for the data sources in Figures 5D to 5F, which are not altered by TMEJ suppression. SV counts are shown above plots for all sample groups.

**G** to **I.** APH-induced junction insertion/microhomology size distributions of the same data sources as Figures 5G to 5I, now normalized to the summed target region coverage in each group. Thus, the y-axis scale of these plots is the same as the SV Frequency plots to reveal the absolute formation efficiency of different SV junction types. SV counts are shown above plots for all sample groups.

**J.** Partial inhibition of deletion SV formation in asynchronous GM12878 cells by ART558. This single replicate experiment matches results from HF1 and HCT116 cells. SV numbers by APH/ART558 are -/-, 42; +/-, 313; +/+, 119.

**K.** APH-induced junction insertion/microhomology size distributions for the data sources for the data sources in panel J.

**L.** The same data as panel K normalized to the summed target region coverage as in panels G to I.

Source data are provided as a Source Data file.

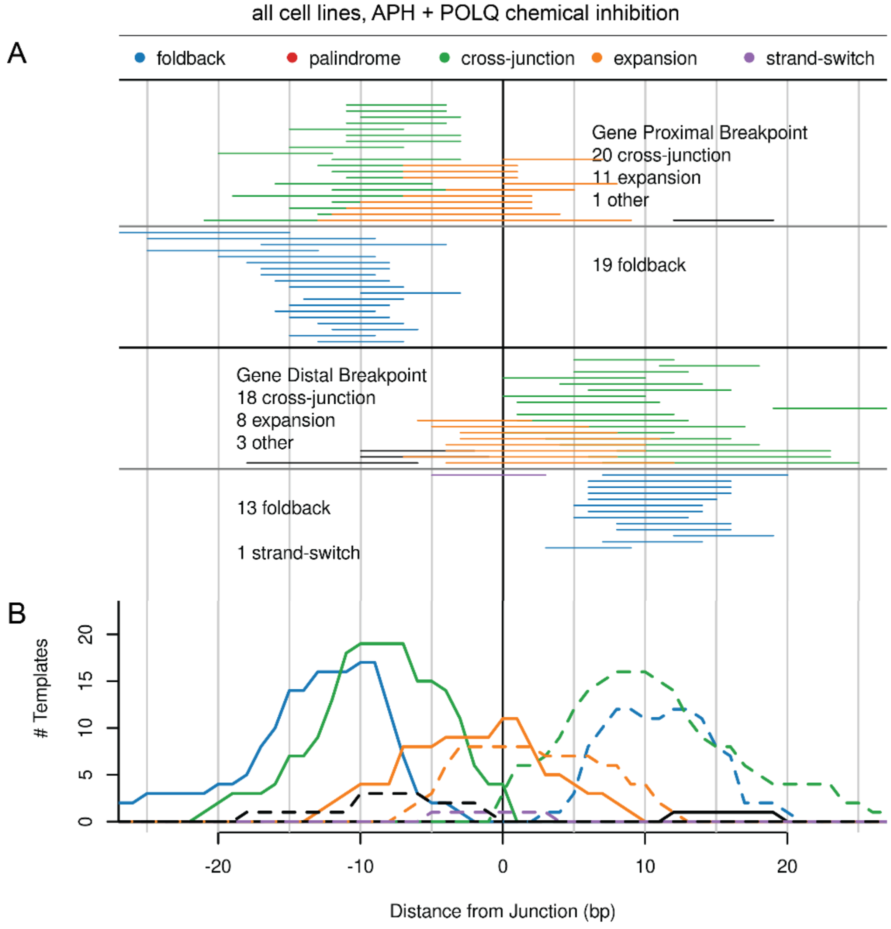


**Supplementary Figure 11. Insertion template locations under POLQ inhibition.**

**A** and **B.** Pileup and histogram plots like Figures 4F and 4G, respectively, showing insertion template locations for all cell lines treated with low-dose APH plus either NVB or ART558 to inhibit POLQ. A larger fraction of templates are of the expansion class under POLQ inhibition, but some cross-junction and foldback templates are observed. 94 SVs are plotted. Source data are provided as a Source Data file.

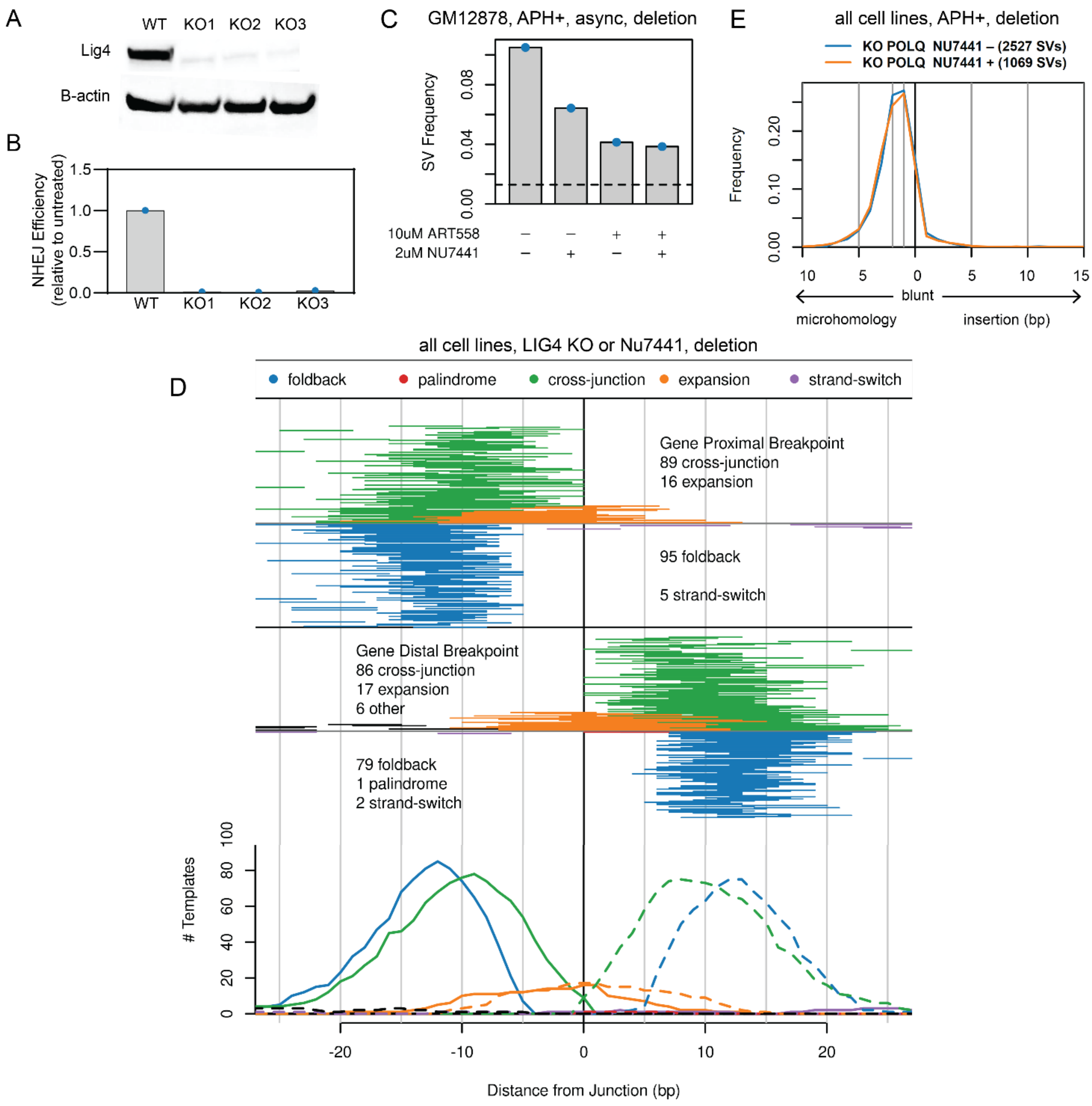


**Supplementary Figure 12. Relationship between TMEJ and NHEJ in SV formation.**

**A** and **B.** Western blot and NHEJ assay with transfected oligonucleotides, respectively, verifying *LIG4* KO in HCT116 cells (n=1 per control and KO line).

**C.** Effect of TMEJ and NHEJ chemical inhibition on deletion formation in APH-treated GM12878 cells (n=1 per treatment combination). The dashed line is the SV frequency observed in non-APH-treated cells. SV counts by ART558/Nu7441 are: -/-, 313; -/+, 180; +/-, 119; +/+, 150.

**D.** Plots showing unaltered insertion template locations in APH-induced deletion SVs from samples lacking NHEJ due to either *LIG4* knockout or treatment with NU7441, as compared to data in Figures 4F and 4G. 396 total insertions SVs are plotted.

**E.** NHEJ loss does not alter junction microhomology and insertion usage in the absence of POLQ. Each trace includes APH-induced deletion SVs across all cell lines and cell cycle stages in *POLQ*$^{-/-}$ cells. Biological replicate (SV) counts by Nu7441 are: -, 9 (2527); +, 8 (1069).

Source data are provided as a Source Data file.
