# Supplementary material for: Replication stress induces POLQ-mediated structural variant formation throughout common fragile sites after entry into mitosis
Source: Nat Commun. 2024 Nov 6;15:9582. doi: 10.1038/s41467-024-53917-8 (PMC11541566; doi:10.1038/s41467-024-53917-8)
Supplement: Supplementary file 2 — Description of Additional Supplementary Files [file 41467_2024_53917_MOESM2_ESM.pdf]

Description of Additional Supplementary Files

File Name: Supplementary Data 1
Description: svCapture samples with project batches, samples names and identifiers, and experimental conditions.

File Name: Supplementary Data 2
Description: Properties of individual SV junctions of all types from all samples.

File Name: Supplementary Data 3
Description: Aggregated experimental results for all groups of replicate samples, deletion SVs only.

File Name: Supplementary Data 4
Description: Excel file with three spreadsheets listing details of (i) CRISPR guide RNAs, (ii) CRISPR KO alleles, and (iii) oligonucleotides used in the TMEJ-NHEJ assay.
