# Supplementary material for: Replication stress induces POLQ-mediated structural variant formation throughout common fragile sites after entry into mitosis
Source: Nat Commun. 2024 Nov 6;15:9582. doi: 10.1038/s41467-024-53917-8 (PMC11541566; doi:10.1038/s41467-024-53917-8)
Supplement: Supplementary file 7 — Reporting Summary [file 41467_2024_53917_MOESM7_ESM.pdf]

nature portfolio

Corresponding author(s): Thomas E. Wilson, Thomas W. Glover

Last updated by author(s): Oct 11, 2024

Reporting Summary

Nature Portfolio wishes to improve the reproducibility of the work that we publish. This form provides structure for consistency and transparency in reporting. For further information on Nature Portfolio policies, see our Editorial Policies and the Editorial Policy Checklist.

Statistics

For all statistical analyses, confirm that the following items are present in the figure legend, table legend, main text, or Methods section.

n/a	Confirmed	
☐	☒	The exact sample size (*n*) for each experimental group/condition, given as a discrete number and unit of measurement
☐	☒	A statement on whether measurements were taken from distinct samples or whether the same sample was measured repeatedly
☐	☒	The statistical test(s) used AND whether they are one- or two-sided<br>*Only common tests should be described solely by name; describe more complex techniques in the Methods section.*
☐	☒	A description of all covariates tested
☐	☒	A description of any assumptions or corrections, such as tests of normality and adjustment for multiple comparisons
☐	☒	A full description of the statistical parameters including central tendency (e.g. means) or other basic estimates (e.g. regression coefficient) AND variation (e.g. standard deviation) or associated estimates of uncertainty (e.g. confidence intervals)
☐	☒	For null hypothesis testing, the test statistic (e.g. *F*, *t*, *r*) with confidence intervals, effect sizes, degrees of freedom and *P* value noted<br>*Give P values as exact values whenever suitable.*
☒	☐	For Bayesian analysis, information on the choice of priors and Markov chain Monte Carlo settings
☒	☐	For hierarchical and complex designs, identification of the appropriate level for tests and full reporting of outcomes
☒	☐	Estimates of effect sizes (e.g. Cohen's *d*, Pearson's *r*), indicating how they were calculated

Our web collection on statistics for biologists contains articles on many of the points above.

Software and code

Policy information about availability of computer code

Data collection: No software was used to collect the data in this study.

Data analysis	We previously reported the svCapture data analysis pipeline and app constructed in the svx-mdi-tools suite of the Michigan Data Interface (MDI). Version 2.0.0 of the tool suite or higher was used for data analysis, contemporary with version 3.0.0 of the genomex-mdi-tools suite dependency and versions 1.3.2 and 1.8.2 of the mdi-pipeline-frameworks and mdi-apps-framework, respectively. Additional program dependency versions are set by the conda environment definitions tied to tool suite versions.<br><br>Code comprising the svCapture data analysis pipeline and app is maintained in the svx-mdi-tools GitHub repository with all releases captured in Zenodo (DOI 10.5281/zenodo.7871676). Data-specific job scripts used to execute the pipeline for samples in this manuscript and associated support files, including resource files, sample lists, and job logs, are maintained in a separate GitHub repository linked to Zenodo (DOI 10.5281/zenodo.10728194). Together, data and code above can reproduce our analyses.<br><br>Additional software used includes:<br>GraphPad Prism 10<br>CHOPCHOP, Labun et al., 2019<br>fastp v0.23.2, Chen et al., 2018<br>bwa mem v0.7.17, Li H., 2013<br>Synthego Performance Analysis, ICE Analysis. 2019. v3.0. Synthego<br>FlowJo v10.8 Software, BD Life Sciences

For manuscripts utilizing custom algorithms or software that are central to the research but not yet described in published literature, software must be made available to editors and reviewers. We strongly encourage code deposition in a community repository (e.g. GitHub). See the Nature Portfolio guidelines for submitting code & software for further information.

Data

Policy information about availability of data

All manuscripts must include a data availability statement. This statement should provide the following information, where applicable:

- Accession codes, unique identifiers, or web links for publicly available datasets
- A description of any restrictions on data availability
- For clinical datasets or third party data, please ensure that the statement adheres to our policy

svCapture sequencing data have been deposited in two repositories. Samples lists are provided as Supplementary Data 1. Data from cell line UM-HF1 require human data access restrictions and were deposited into the Database of Genotypes and Phenotypes (dbGaP, accession phs003121.v2.p1 [https://www.ncbi.nlm.nih.gov/projects/gap/cgi-bin/study.cgi?study_id=phs003121.v2.p1]) and are available without further restrictions by registering a request with dbGaP. Data from commercially available cell lines HCT116 and GM12878 were deposited into the public Sequence Read Archive (SRA, BioProject ID PRJNA1085257 [https://www.ncbi.nlm.nih.gov/bioproject/PRJNA1085257]). Flow cytometry data were deposited into Mendeley Data (DOI 10.17632/mz53d2486n.1 [https://doi.org/10.17632/mz53d2486n.1]). The main processed data outputs of the svCapture pipeline are provided as Supplementary Data 2 and 3, included in the Zenodo code set linked to GitHub alongside the job scripts that generated them (see below), or in a separate Zenodo dataset carrying larger output files (DOI 10.5281/zenodo.10916986 [https://doi.org/10.5281/zenodo.10916986]), including data packages and app bookmarks. Source data are provided with this paper.

Research involving human participants, their data, or biological material

Policy information about studies with human participants or human data. See also policy information about sex, gender (identity/presentation), and sexual orientation and race, ethnicity and racism.

Reporting on sex and gender	*Use the terms sex (biological attribute) and gender (shaped by social and cultural circumstances) carefully in order to avoid confusing both terms. Indicate if findings apply to only one sex or gender; describe whether sex and gender were considered in study design; whether sex and/or gender was determined based on self-reporting or assigned and methods used.*<br>*Provide in the source data disaggregated sex and gender data, where this information has been collected, and if consent has been obtained for sharing of individual-level data; provide overall numbers in this Reporting Summary. Please state if this information has not been collected.*<br>*Report sex- and gender-based analyses where performed, justify reasons for lack of sex- and gender-based analysis.*
Reporting on race, ethnicity, or other socially relevant groupings	*Please specify the socially constructed or socially relevant categorization variable(s) used in your manuscript and explain why they were used. Please note that such variables should not be used as proxies for other socially constructed/relevant variables (for example, race or ethnicity should not be used as a proxy for socioeconomic status).*<br>*Provide clear definitions of the relevant terms used, how they were provided (by the participants/respondents, the researchers, or third parties), and the method(s) used to classify people into the different categories (e.g. self-report, census or administrative data, social media data, etc.)*<br>*Please provide details about how you controlled for confounding variables in your analyses.*
Population characteristics	*Describe the covariate-relevant population characteristics of the human research participants (e.g. age, genotypic information, past and current diagnosis and treatment categories). If you filled out the behavioural & social sciences study design questions and have nothing to add here, write "See above."*
Recruitment	*Describe how participants were recruited. Outline any potential self-selection bias or other biases that may be present and how these are likely to impact results.*
Ethics oversight	*Identify the organization(s) that approved the study protocol.*

Note that full information on the approval of the study protocol must also be provided in the manuscript.

Field-specific reporting

Please select the one below that is the best fit for your research. If you are not sure, read the appropriate sections before making your selection.

☒ Life sciences ☐ Behavioural & social sciences ☐ Ecological, evolutionary & environmental sciences

For a reference copy of the document with all sections, see nature.com/documents/nr-reporting-summary-flat.pdf

Life sciences study design

All studies must disclose on these points even when the disclosure is negative.

Sample size	Sample sizes were not predetermined or calculated in advance. Replicate experiments were performed until statistical difference between critical sample comparisons was established or refuted.
Data exclusions	No individual data points were excluded from the reported data sets, in particular all samples from all reported svCapture library batches are included on plots and in tables.
Replication	All experiments in the main figures were replicated in at least two, typically three or more independent experiments. High sequencing costs sometimes constrained the number of replicates that could be performed. In particular, a few analyses were only performed once for a given combination of experimental parameters. We choose to report these as supplementary information noting that the results are unreplicated but consistent with other cell lines where replicate analysis was performed.
Randomization	Not applicable to this study which applies prospective experimental manipulations of cultured cell lines.
Blinding	Initial scoring of cytogenetic analyses was performed by a scorer blinded to sample identities. Blinded was generally not possible and not required for svCapture analysis because scoring metrics are objective, as determined by the computational algorithms applied the same way to all samples and not subject to individual interpretation.

Reporting for specific materials, systems and methods

We require information from authors about some types of materials, experimental systems and methods used in many studies. Here, indicate whether each material, system or method listed is relevant to your study. If you are not sure if a list item applies to your research, read the appropriate section before selecting a response.

Materials & experimental systems

n/a	Involved in the study	
☐	☒	Antibodies
☐	☒	Eukaryotic cell lines
☒	☐	Palaeontology and archaeology
☒	☐	Animals and other organisms
☒	☐	Clinical data
☒	☐	Dual use research of concern
☒	☐	Plants

Methods

n/a	Involved in the study	
☒	☐	ChIP-seq
☐	☒	Flow cytometry
☒	☐	MRI-based neuroimaging

Antibodies

Antibodies used	Phospho-Histone H3, Cell Signaling Technology #3465<br>Cleaved Caspase-3, Cell Signaling Technology #9602
Validation	Antibody specificity is evident by the expected loss of target protein bands in knockout clones and by the expected pattern of alterations in cell frequencies in gated populations of treated cells following manipulations such as colchicine or etoposide treatment.

Eukaryotic cell lines

Policy information about cell lines and Sex and Gender in Research

Cell line source(s)	UM-HF1 (abbreviated HF1 throughout) is a XY male, euploid, TERT-immortalized human foreskin-derived fibroblast cell line derived and maintained at the University of Michigan subject to human data access restrictions.<br><br>GM12878, (Coriell, RRID CVCL_7526), is a highly studied XX female, euploid, EBV-immortalized human lymphoblastoid cell line generated as part of the HapMap Project.<br><br>HCT116 (ATCC, RRID CVCL_0291) is a highly studied male, mismatch-repair deficient, human colon cancer cell line.

Authentication	Cells lines were analyzed as part of this study by repeated karyotyping and DNA sequencing, which provided ongoing validation of their identity by characteristic genetic variants found in the cell lines.
Mycoplasma contamination	All cell lines were assayed for Mycoplasma and were negative.
Commonly misidentified lines (See ICLAC register)	No cell lines used in this study are listed as commonly misidentified.

Plants

Seed stocks	*Report on the source of all seed stocks or other plant material used. If applicable, state the seed stock centre and catalogue number. If plant specimens were collected from the field, describe the collection location, date and sampling procedures.*
Novel plant genotypes	*Describe the methods by which all novel plant genotypes were produced. This includes those generated by transgenic approaches, gene editing, chemical/radiation-based mutagenesis and hybridization. For transgenic lines, describe the transformation method, the number of independent lines analyzed and the generation upon which experiments were performed. For gene-edited lines, describe the editor used, the endogenous sequence targeted for editing, the targeting guide RNA sequence (if applicable) and how the editor was applied.*
Authentication	*Describe any authentication procedures for each seed stock used or novel genotype generated. Describe any experiments used to assess the effect of a mutation and, where applicable, how potential secondary effects (e.g. second site T-DNA insertions, mosiacism, off-target gene editing) were examined.*

Flow Cytometry

Plots

Confirm that:

☒ The axis labels state the marker and fluorochrome used (e.g. CD4-FITC).

☒ The axis scales are clearly visible. Include numbers along axes only for bottom left plot of group (a 'group' is an analysis of identical markers).

☒ All plots are contour plots with outliers or pseudocolor plots.

☒ A numerical value for number of cells or percentage (with statistics) is provided.

Methodology

Sample preparation	Detailed methods for cell preparation for flow cytometry are provided in Methods.
Instrument	Samples were submitted to the University of Michigan Flow Cytometry Core for collecting cell cycle fractions using a FACS Aria III (BD Bioscience) or Bigfoot Cell Sorter (ThermoFisher).
Software	FlowJo v10.8 Software, BD Life Sciences
Cell population abundance	No additional validation of cell purity in gated fractions was performed.
Gating strategy	Cell cycle distributions were first assessed using an asynchronous population to establish gating for the different DNA contents and then applied to synchronized or arrested samples. M-phase cell populations were consistently well separated from G2 cells on the phosphohistone H3 axis as shown in example 2D plots.

☒ Tick this box to confirm that a figure exemplifying the gating strategy is provided in the Supplementary Information.
