# Supplementary figures and images for: Replication stress induces POLQ-mediated structural variant formation throughout common fragile sites after entry into mitosis
Source: Nat Commun. 2024 Nov 6;15:9582. doi: 10.1038/s41467-024-53917-8 (PMC11541566; doi:10.1038/s41467-024-53917-8)

Wilson et al.

Uncropped gel images

Figure S12A

B-Actin Western

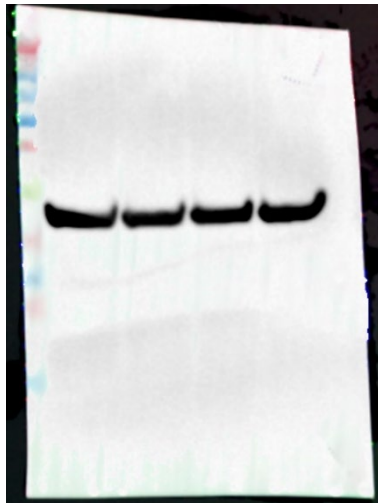

LIG4 Western

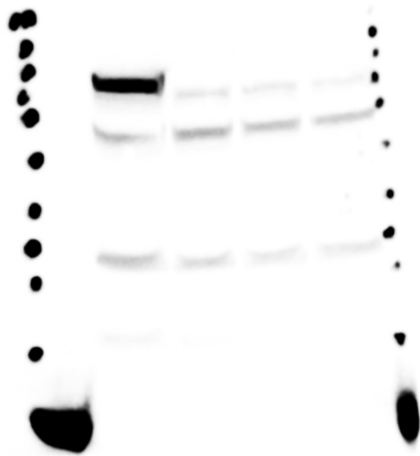

Supplement: Supplementary file 9 — Source Data [file 41467_2024_53917_MOESM9_ESM.zip › Supp_Fig12/Sugg_Fig12A.pdf]
